# Capturing learning persistence through fractional dynamic modeling: evidence from secondary school students

**DOI:** 10.3389/fpsyg.2026.1922588

**Published:** 2026-09-21

**Authors:** Can Wang, Zheng Pang, Shenghan Wang, Guoyu Niu

**Affiliations:** 1 Shandong Second Medical University, Weifang, Shandong, China; 2 Tianjin University, Tianjin, China

**Keywords:** educational memory, educational support and learning behavior variables, fractional dynamic modeling, learning persistence, student academic performance

## Abstract

**Background:**

Student academic performance develops through cumulative learning experiences, yet conventional educational performance models generally assume that Educational support and learning behavior variables produce immediate effects. This study proposes a fractional dynamic framework to capture the persistence of learning and examine associations between educational support, learning behavior, and academic performance.

**Methods:**

We analyzed micro-level data from 649 Portuguese secondary school students using a fractional-order educational performance model that incorporated educational memory through a fractional formulation. The analysis examined associations between school study support, paid academic assistance, family educational support, attendance behavior, and study time and final academic performance while controlling for academic history and socioeconomic characteristics.

**Results:**

The proposed fractional model outperformed the benchmark specification by increasing the adjusted coefficient of determination from 0.849 to 0.886 and reducing the root mean square error from 1.04 to 0.87. The estimated fractional-order parameter (α = 0.83) indicated temporal dependence within the observed academic assessment structure. Family educational support and weekly study time were positively associated with academic performance, whereas student absences and previous academic failures were negatively associated with final achievement.

**Conclusion:**

The findings indicate that incorporating educational memory provides a useful representation of temporal dependence in student academic performance. The observed associations between educational support, learning behavior, and achievement provide descriptive evidence that may inform future longitudinal and intervention-based research.

## 1 Introduction

Academic achievement is widely regarded as one of the most important indicators of educational quality because it reflects the effectiveness of teaching, learning, and institutional support (Huang et al., 2025; Li et al., 2021). Student performance influences educational attainment, employability, psychological wellbeing, and long-term socioeconomic development. Consequently, identifying factors associated with higher academic performance has become a central objective of educational psychology, learning analytics, and educational policy (Cao et al., 2025; Engida et al., 2024; Liu et al., 2022). Recent advances in educational data analysis have enabled researchers to examine how individual characteristics, family environments, and school interventions jointly influence student learning outcomes (Chu et al., 2025; Li and Xue, 2023; Tu et al., 2026). At the same time, the increasing availability of micro-level educational datasets has encouraged the development of quantitative models to support evidence-based educational decision-making (Fei and Wang, 2026; Zhang et al., 2026). Educational data mining and learning analytics have consequently become important research areas for understanding complex learning behaviors and improving educational systems (Fei and Jin, 2026; Vulic et al., 2024).

Educational support and learning behavior variables are among the principal mechanisms through which schools seek to improve student achievement (Jiang et al., 2022; Tang et al., 2026). These interventions include academic support programs, attendance monitoring, individualized learning assistance, parental engagement initiatives, and structured study guidance (Guo et al., 2024; Shahzad et al., 2023). Their primary objective is to reduce learning inequalities while improving students’ academic competence and motivation. Previous research has consistently reported positive associations between sustained educational support and academic achievement, although the magnitude of these effects varies across educational contexts, student characteristics, and intervention types. More importantly, evidence increasingly suggests that the effectiveness of Educational support and learning behavior variables depends upon their continuity rather than isolated implementation, indicating that academic improvement is a cumulative process that develops over time rather than an immediate response to individual educational activities (Cabral-Gouveia et al., 2023; Gordon and Hart, 2022).

Family involvement represents another important determinant of educational success. Parents influence learning through educational expectations, emotional encouragement, supervision of learning, communication with teachers, and the provision of educational resources. Recent meta-analyses have confirmed that parental involvement remains positively associated with academic achievement across different educational systems and subject areas. However, the strength of this relationship depends on the nature of parental engagement. Educational expectations, communication, and encouragement generally produce stronger effects than direct homework assistance alone. These findings indicate that sustained family engagement contributes to the development of motivation to learn and self-regulation, thereby supporting continuous academic progress rather than isolated improvements in examination performance (Kantova, 2024; Pusztai et al., 2023).

Student learning behavior also contributes substantially to academic achievement. Regular attendance, effective study habits, self-management, and educational aspirations collectively influence students’ opportunities to acquire knowledge and develop cognitive skills. Students who maintain consistent attendance and devote sufficient time to independent learning generally demonstrate superior academic performance because repeated exposure to educational activities reinforces knowledge acquisition and retention. Conversely, frequent absences disrupt continuity of learning and often lead to cumulative knowledge deficits that become increasingly difficult to overcome in subsequent learning stages. Recent empirical evidence also highlights the importance of self-regulation and self-efficacy in strengthening the relationship between learning behaviors and educational achievement (Yu et al., 2026).

### 1.1 Literature review

Student academic performance has been extensively examined in educational psychology, learning analytics, and educational management due to its importance for individual development and educational quality. Existing studies consistently identify Educational support and learning behavior variables, parental involvement, attendance, and learning behaviors as important determinants of academic achievement. Nevertheless, previous investigations differ considerably in their methodological approaches and often provide inconsistent evidence regarding the persistence of Associations. Most importantly, most empirical studies estimate static relationships and rarely consider the cumulative nature of student learning.

Educational support and learning behavior variables have been widely investigated as mechanisms for improving learning outcomes. Korpershoek et al. (2025) reported that structured classroom management and instructional interventions significantly improve both student engagement and academic achievement when implemented consistently over time rather than through isolated instructional activities. The authors emphasized that intervention quality and implementation continuity determine the magnitude of educational improvement (Korpershoek et al., 2025). Similarly, Geduld (2024) demonstrated that intervention programs designed to strengthen parental participation enhanced students’ motivation to learn, self-regulation, and academic performance. Although both studies confirmed the positive contribution of Educational support and learning behavior variables, they primarily evaluated immediate educational outcomes without explicitly modeling the persistence of learning across successive educational stages (Geduld, 2024).

Parental involvement remains one of the most extensively investigated determinants of educational achievement. Hannon and O’Donnell (2022) classified parental involvement into several dimensions and demonstrated that students whose parents maintained high educational engagement achieved significantly better academic outcomes than those with limited family participation (Hannon and O’Donnell, 2022). Likewise, Jeynes (2024) conducted a comprehensive meta-analysis and concluded that parental involvement has a significant positive effect on students’ academic performance across educational systems (Jeynes, 2024). More recently, Kim (2022) further demonstrated that parental educational expectations and parental support produce stronger educational benefits than direct homework supervision, indicating that motivational support contributes more effectively to long-term academic development (Kim, 2022). These findings collectively suggest that parental involvement influences academic achievement through multiple psychological and educational mechanisms rather than through direct instructional assistance alone.

Although previous studies consistently support the importance of family engagement, differences remain regarding the mechanisms through which parental involvement affects learning outcomes. Chen and Mok (2023) argued that students’ perceptions of parental academic involvement positively influence academic adaptability and resilience by strengthening mastery goal orientation (Chen and Mok, 2023). Similarly, Geduld (2024) reported that parental involvement promotes self-efficacy and academic confidence, which subsequently improve educational achievement. In contrast, Kim (2022) observed that excessive parental control may reduce the effectiveness of educational support when parental involvement limits student autonomy. These findings indicate that parental involvement should be evaluated not only by its frequency but also by its educational quality and psychological characteristics.

Attendance behavior has likewise received considerable attention within the educational literature because regular classroom participation directly influences opportunities for knowledge acquisition. Zhou et al. (2023) demonstrated that consistent attendance strongly predicts subsequent academic achievement and further reported that attendance behavior remains relatively stable throughout the academic semester (Zhou et al., 2023). Their analysis also revealed that attendance patterns exhibit important peer effects, suggesting that learning behavior develops within broader social environments. More recent intervention studies have similarly reported that attendance support programs improve both school participation and educational outcomes, although their analyses primarily evaluate behavioral improvements instead of cumulative learning processes.

Study behavior represents another important determinant of student achievement. Previous investigations consistently report that students who devote greater effort to independent learning generally achieve higher academic performance because repeated study reinforces knowledge acquisition and retention. Recent studies in educational psychology further indicate that self-regulation, self-efficacy, and learning motivation mediate the relationship between study behavior and academic achievement (Drugova et al., 2024). These findings complement attendance research by demonstrating that successful learning depends not only on classroom participation but also on students’ continuous engagement with educational activities beyond formal instruction. Consequently, learning behavior should be regarded as a dynamic process that develops progressively throughout the educational experience rather than as a collection of isolated academic activities.

Recent developments in learning analytics have introduced increasingly sophisticated approaches for predicting educational outcomes. Machine learning techniques, including random forests, gradient boosting, artificial neural networks, and deep learning algorithms, frequently outperform conventional regression models in predictive accuracy. However, these approaches primarily emphasize prediction rather than explanation and often operate as complex computational models with limited theoretical interpretation. Furthermore, most learning analytics studies estimate relationships using cross-sectional observations and therefore provide limited insight into how previous learning experiences influence future academic achievement. Consequently, the temporal persistence of Educational support and learning behavior variables remains insufficiently represented despite substantial improvements in predictive performance.

A common characteristic of the existing literature is the reliance on conventional regression models, structural equation modeling, multilevel analysis, or machine learning algorithms that implicitly assume immediate responses of academic achievement to educational inputs. Although these methods successfully identify statistically significant determinants of student performance, they rarely account for educational memory or cumulative learning. Previous academic achievement is generally treated as an ordinary explanatory variable rather than as the dynamic accumulation of prior educational experiences. As a result, delayed educational responses and long-term persistence remain largely unexplored within existing empirical models (Rabelo et al., 2024).

The above discussion reveals three important research gaps. First, previous studies predominantly investigate Educational support and learning behavior variables using static modeling approaches that neglect educational memory. Second, Educational support and learning behavior variables, attendance behavior, family educational support, and previous academic achievement are rarely integrated within a unified dynamic modeling framework. Third, despite the growing use of fractional dynamic modeling in engineering, economics, epidemiology, and biological systems, its use in educational psychology remains extremely limited. The present study addresses these limitations by developing a fractional dynamic framework that explicitly incorporates memory dependence in modeling student academic performance. By integrating Educational support and learning behavior variables with accumulated learning experiences, the proposed approach provides a more comprehensive representation of the dynamic processes underlying academic achievement than conventional models of educational performance.

Despite the substantial body of research examining Educational support and learning behavior variables, most empirical studies rely on conventional statistical models that implicitly assume educational outcomes respond immediately to current explanatory variables. Linear regression, generalized linear models, and many machine learning approaches primarily estimate contemporaneous relationships while paying limited attention to the persistence of prior learning. Such approaches may adequately describe short-term associations but provide only limited insight into the cumulative nature of learning. Knowledge acquisition rarely occurs instantaneously. Instead, new knowledge is integrated with previously acquired information through continuous practice, reinforcement, and cognitive development. Consequently, current academic performance often reflects both present educational conditions and accumulated educational experiences.

Educational psychology provides strong theoretical support for this cumulative interpretation of learning. Cognitive learning theories suggest that knowledge is progressively constructed through repeated interaction with previous experiences, whereas social learning perspectives emphasize the continuous influence of family, school, and environmental factors on behavioral development. Similarly, theories of self-regulated learning propose that repeated use of learning strategies strengthens academic competence through continuous adaptation and reflection. Collectively, these theoretical perspectives indicate that student achievement is persistent, suggesting that present academic performance depends on both current Educational support and learning behavior variables and prior learning experiences (Hanham et al., 2023; Sweller, 2022).

Fractional calculus provides a mathematical framework capable of representing such persistence through the concept of memory dependence. Unlike conventional integer-order derivatives, fractional derivatives incorporate information from previous states of a dynamic process, thereby allowing present system behavior to depend on accumulated historical observations. Fractional dynamic models have been successfully applied in engineering, biological sciences, economics, epidemiology, and finance because they provide more realistic descriptions of systems characterized by long-term dependence and delayed responses. Their application within educational psychology, however, remains extremely limited despite the inherently cumulative nature of learning and cognitive development.

The limited integration of fractional dynamic modeling into educational research represents an important methodological gap. Existing educational studies frequently evaluate Predictive or explanatory performance using static or short-term approaches without explicitly modeling the persistence of learning. Consequently, the delayed effects of educational support, attendance behavior, family engagement, and prior academic experiences may not be adequately captured by conventional educational performance models. Incorporating educational memory into quantitative modeling, therefore, offers an opportunity to improve both theoretical understanding and empirical prediction of student achievement.

The present study addresses this gap by developing a fractional dynamic framework to model students’ academic performance in response to Educational support and learning behavior variables. Using micro-level educational data from Portuguese secondary school students, the proposed framework integrates Educational support and learning behavior variables, family characteristics, learning behavior, and prior academic achievement into a fractional-order specification that explicitly represents educational memory. Unlike conventional educational performance models, the proposed approach assumes that current academic achievement is influenced by accumulated prior learning experiences and current educational conditions.

This study makes three principal contributions to the educational psychology literature. First, it introduces fractional dynamic modeling as a novel analytical framework for investigating student academic performance. Second, it extends existing educational performance models by incorporating educational memory into the learning process via a fractional-order formulation. Third, it provides empirical evidence regarding the cumulative effects of Educational support and learning behavior variables and demonstrates the potential value of fractional dynamic modeling for supporting evidence-based educational planning and long-term intervention strategies. The methodological framework developed in this study, therefore, contributes to both educational psychology and learning analytics by providing a more comprehensive representation of the dynamic processes underlying student academic achievement.

## 2 Methodology

### 2.1 Research framework

This study proposes a fractional dynamic framework to examine the temporal association between educational support, learning behavior, and student academic performance. The framework is based on the premise that learning is a cumulative process in which current academic outcomes depend not only on present educational inputs but also on the persistence of previous learning experiences. Conventional educational models generally assume that interventions produce immediate effects and that past influences diminish rapidly. Such assumptions may overlook the gradual nature of knowledge acquisition and the lasting impact of educational support. To address this limitation, fractional calculus is incorporated to capture memory dependence in the learning process.

The theoretical motivation for the fractional formulation assumes that the influence of previous learning experiences does not disappear immediately after an assessment. Earlier learning may retain a decreasing influence on subsequent performance, with more recent experiences expected to have greater weight than distant experiences. The Caputo formulation provides a mathematical representation of this type of fading memory through a kernel that assigns decreasing weights to previous states. The use of a fractional derivative therefore does not imply that learning is proven to follow a power law. Instead, it provides a parsimonious candidate representation of gradual memory decay that can be examined empirically through the estimated fractional order α. Within this interpretation, α controls the form and strength of the memory weighting in the fractional representation. Values of α closer to 1 correspond to behavior closer to the conventional first-order formulation, whereas values below 1 represent a fractional specification with a noninteger memory structure. Thus, α is interpreted as a parameter describing the degree of fractional temporal dependence in the observed academic trajectory rather than as a direct psychological measure of memory.

The proposed framework considers student performance as a function of several educational support, family, and learning behavior variables observed in the dataset. These interventions include structured study support, attendance monitoring, parental engagement, and other student and family characteristics. These observed factors are specified as explanatory variables in the empirical model. Their estimated coefficients describe conditional associations with academic achievement and should not be interpreted as causal effects. The fractional-order derivative represents this accumulation by allowing historical educational experiences to influence present learning outcomes. Consequently, the model provides a more realistic description of educational dynamics than classical integer-order models.

The analytical framework consists of four sequential stages. First, micro-level student data are collected, and relevant educational variables are identified. Second, Explanatory variables and control variables are constructed from the dataset. Third, the fractional dynamic model is specified to examine conditional associations between the observed educational and behavioral factors and student performance while representing temporal dependence through the fractional component. Finally, model estimation and robustness analyses are conducted to evaluate the effectiveness of the proposed framework and to compare its performance with conventional approaches.

Figure 1 illustrates the overall research framework adopted in this study. The process begins with collecting student-level educational data and selecting variables representing academic support, attendance, parental involvement, and demographic characteristics. These variables are then incorporated into the fractional dynamic model, where the memory parameter captures the persistence of past Educational support and learning behavior variables. The estimated model produces measures of academic performance that are subsequently evaluated through robustness tests and comparisons with classical dynamic models. The framework, therefore, provides an integrated approach for examining the cumulative effects of Educational support and learning behavior variables and their implications for student achievement.

**FIGURE 1:**
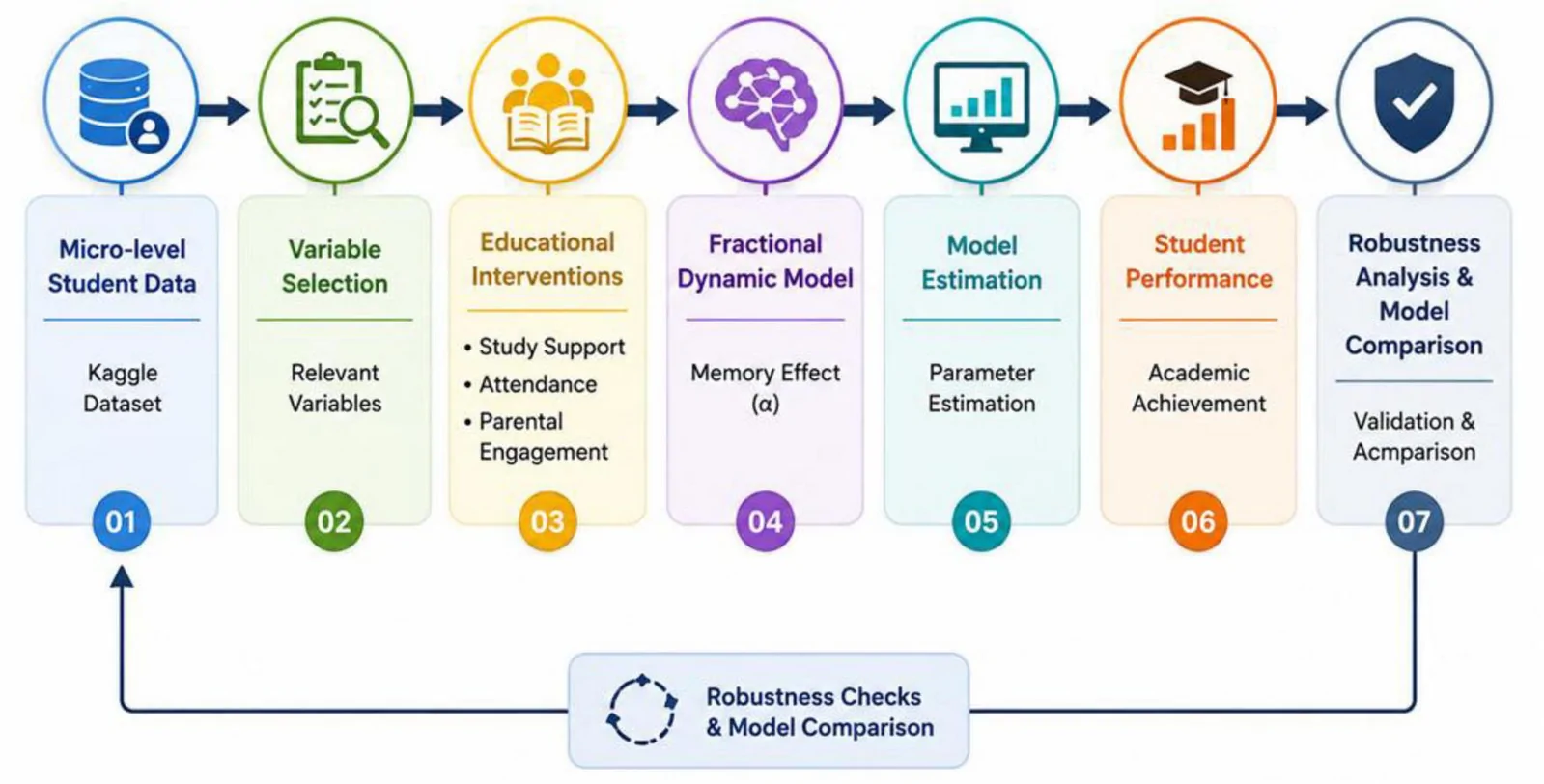
Proposed research framework for the fractional dynamic modeling of student performance under Educational support and learning behavior variables.

### 2.2 Model specification

The proposed model is specified to investigate how Educational support and learning behavior variables influence student academic performance while accounting for the persistence of learning outcomes. The model assumes that academic achievement is determined by a combination of institutional support, family characteristics, and individual learning behaviors. These factors are treated as interconnected determinants rather than independent influences because the learning process is cumulative and develops through repeated educational experiences.

Figure 2 presents the hierarchical structure of the proposed model specification. Student academic performance, measured by the final course grade (G3), is specified as the dependent variable. The explanatory variables are selected according to the conceptual framework presented in Figure 2. Educational support and learning behavior variables include school study support, additional paid academic assistance, family educational support, student absences, and weekly study time. The control variables include previous academic performance, prior academic failures, family educational background, educational aspirations, internet access, and family relationship quality. Mother’s education, father’s education, and family relationship quality are retained as indicators of family background.

**FIGURE 2:**
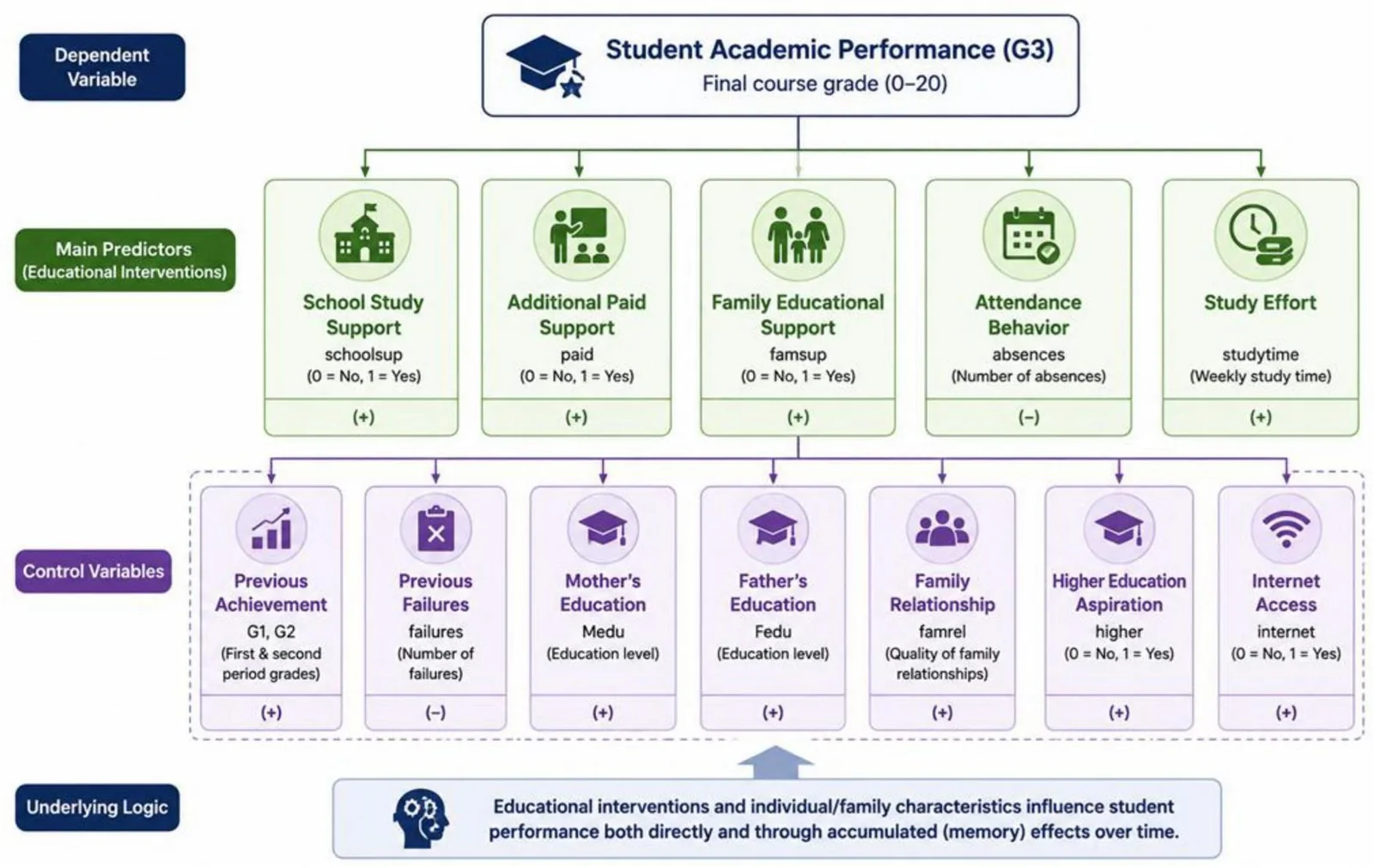
Hierarchical structure of the proposed model specification.

The second group comprises control variables that account for differences in students’ educational background and personal circumstances. Previous academic achievement is represented by the first- and second-period grades, while previous academic failures reflect accumulated learning difficulties. Parental education is included as a proxy for family socioeconomic and educational resources, whereas family relationship quality reflects the home learning environment. Higher education aspirations and internet access are included because they indicate students’ educational motivation and their access to learning resources beyond the classroom. These variables are included to account for observed differences in students’ academic history, family background, aspirations, and access to learning resources. Their inclusion reduces the risk of omitted observed heterogeneity, although residual confounding cannot be excluded.

As illustrated in Figure 2, the hierarchical organization distinguishes between variables that directly represent Educational support and learning behavior variables and variables that characterize students’ individual and family backgrounds. This classification provides a structured representation of the educational system and clarifies the conceptual role of each variable within the empirical model. Educational support and learning behavior variables are the primary variables of interest, whereas the remaining variables serve to control for student heterogeneity.

Unlike conventional educational performance models, the proposed specification assumes that the influence of Educational support and learning behavior variables is not restricted to the current learning period. Educational support, attendance, and study behavior generate cumulative effects that persist over time through knowledge retention and repeated learning experiences. This characteristic suggests that student performance is memory-dependent and cannot be adequately represented by classical integer-order models. Consequently, the model is extended through fractional calculus to incorporate the persistence of previous educational experiences into the dynamic learning process. The mathematical formulation of the fractional dynamic model is presented in the following subsection.

### 2.3 Fractional dynamic educational model

The hierarchical specification presented in Figure 2 provides the conceptual foundation for the mathematical model developed in this study. Student academic performance is assumed to evolve as a function of Educational support and learning behavior variables and individual characteristics. To establish the proposed framework, the analysis begins with the conventional educational performance model and then extends it using fractional calculus. The general educational performance function is expressed in Equation (1) as


P⁢(t)=f⁢(E⁢I⁢(t),C⁢V⁢(t)) (1)

where (P(t)) denotes student academic performance, (EI(t)) represents the vector of educational Explanatory variables, and (CV(t)) denotes the vector of control variables.

The educational support and learning behavior vector is defined in Equation (2) as


E⁢I⁢(t)=[S⁢S,P⁢A,F⁢S,A⁢T,S⁢T] (2)

SS denotes school study support, PA denotes additional paid academic assistance, FS denotes family educational support, AT denotes student attendance behavior measured by the number of absences, and ST denotes weekly study time. Similarly, the control variable vector is specified in Equation (3) as


CV(t)=[PG(t),PF(t),ME(t),FE(t),FR(t),HE(t),IA(t) (3)

where (PG) denotes previous academic achievement, (PF) represents previous academic failures, (ME) and (FE) denote the educational attainment of the mother and father, respectively, (FR) represents family relationship quality, (HE) indicates higher education aspiration, and (IA) denotes internet access. The conventional observational performance model is expressed as:


P⁢(t)=β⁢0+β⁢1⁢S⁢S+β⁢2⁢P⁢A+β⁢3⁢F⁢S+β⁢4⁢A⁢T+β⁢5⁢S⁢T (4)

  +γ⁢CV′+ε⁢(t)

where β_0_ denotes the intercept; β_1_, β_2_, β_3_, β_4_, and β_5_ represent the regression coefficients for SS, PA, FS, AT, and ST, respectively; γ denotes the vector of coefficients for the control variables (CV); and ε(t) denotes the random error term.

Equation (4) assumes that Educational support and learning behavior variables influence student performance only within the current learning period. However, educational psychology suggests that knowledge acquisition is cumulative and that previous learning experiences continue to affect subsequent academic achievement. To account for this persistence, the model is extended through the Caputo fractional derivative. The Caputo fractional derivative of order 0 < α < 1 is defined in Equation (5) as:


$$C\,D_{t}^{\alpha }\,f\,\left(t\right)=\frac{1}{\Gamma \,\left(1-\alpha \right)}\,\int_{0}^{t}\frac{f^{{}^{\prime }}\,\left(\tau \right)}{{\left(t-\tau \right)}^{\alpha }}\,d\tau$$ (5)

For empirical implementation, the three academic assessments available in the dataset, G1, G2, and G3, are treated as ordered observations of student academic performance within the academic year. G1 represents the first assessment period, G2 the second, and G3 the final. The fractional memory component is therefore constructed from these three ordered observations rather than from a conventional cross-sectional representation of previous grades. Because only three assessment points are available for each student, the empirical fractional term provides a limited discrete representation of temporal dependence. It does not fully resolve the long-memory trajectory.

Using the Caputo fractional derivative (Caputo, 1967), the proposed model is formulated in Equation (6) as:


$$C\,D_{t}^{\alpha }\,P\,\left(t\right)=\beta_{0}+\beta_{1}\,S\,S+\beta_{2}\,P\,A+\beta_{3}\,F\,S+\beta_{4}\,A\,T$$ (6)

$$\;\;+\beta_{5}\,S\,T+\gamma^{{}^{\prime }}\,C\,V+\varepsilon \,\left(t\right)$$

Equation (6) is operationalized at the final assessment period by using a discrete approximation of the Caputo derivative. The three academic assessments are represented by equally spaced temporal points, with G1, G2, and G3 corresponding to the first, second, and final assessment periods, respectively. A two-interval discrete Caputo approximation is used to evaluate the fractional derivative at G3. Under this specification, the fractional derivative is calculated from the change between G2 and G3 together with the weighted change between G1 and G2. The predicted value of G3 is then obtained by rearranging the discrete fractional equation so that G3 is expressed as a function of G1, G2, the explanatory variables, the regression parameters, and the fractional order parameter. Thus, Equation (6) is converted into an individual predicted academic performance value before the residual sum of squares is calculated. The temporal step is denoted by Δt and is defined as one assessment interval. Because the dataset does not include exact calendar dates, the three assessment periods are represented by the normalized temporal grid t1 = 0, t2 = 1, and t3 = 2. Consequently, the temporal step is set to one normalized assessment unit. The discrete approximation follows the standard numerical treatment of Caputo fractional derivatives, in which previous states contribute to the fractional derivative through order-dependent weights (Caputo, 1967).


$${\text{D}}^{\text{ca}}\,P\,\left(t_{3}\right)=$$ (6a)

$$\left[\left(G_{3}-G_{2}\right)+\left(2^{1-a}-1\right)\,\left(G_{2}-G_{1}\right)\right]/\left[\Gamma \,\left(2-\alpha \right)\,\Delta \,{\text{t}}^{\text{a}}\right]$$

Substitution of the discrete approximation into Equation (6) gives the predicted final academic performance in the form:


$${\hat{G}}_{3}=G_{2}+\Gamma \left(2-\alpha \right)\Delta {\text{t}}^{a}[\beta_{0}+\beta_{1}\text{SS}+\beta_{2}\text{PA}+\beta_{3}\text{FS}$$ (6b)

+β4AT+β5ST+γCV′]-(21-a-1)(G2-G1)

G1 provides the initial condition, representing the first observed academic performance state. G2 provides the intermediate state required for the fractional memory term, and G3 is the observed outcome used for model evaluation. Therefore, each student contributes one outcome prediction based on the available academic history G1 and G2. The initial condition is defined by the first academic assessment, G1. The second assessment, G2, provides the intermediate academic state required by the fractional approximation, whereas G3 represents the observed final academic outcome. No spatial boundary condition is required because the model describes academic performance over discrete assessment periods rather than a spatial process.

The discrete approximation follows the L1 formulation of the Caputo derivative on a uniform temporal grid. The three assessment points are defined as t_1_ = 0, t_2_ = 1, and t_3_ = 2, so that Δt = 1 represents one assessment interval. The fractional derivative at the final assessment is therefore calculated from the two available academic transitions, G1 to G2 and G2 to G3. The resulting value is substituted into the fractional model, and Equation (6b) provides the predicted final grade for each student. The initial condition is defined by the first observed academic performance, G1. No external boundary condition is imposed because the model is formulated over a temporal academic sequence rather than a spatial domain. G2 provides the intermediate observation required by the discrete fractional approximation, and G3 is used as the observed outcome.

The empirical estimation of Equation (6) enables the examination of conditional associations between the observed explanatory variables and final academic performance while representing temporal dependence through the fractional memory component. The estimated coefficients should not be interpreted as causal intervention effects because the data are observational.

### 2.4 Estimation procedure

The proposed fractional dynamic model is estimated using the student-level observations obtained from the educational dataset. The temporal component is constructed from the ordered academic assessments G1, G2, and G3. G1 is treated as the initial observation, G2 as the intermediate observation, and G3 as the final observation. The fractional memory term is calculated from the changes between these successive academic assessments. Thus, G1 and G2 provide the historical academic information required for the fractional component, while G3 represents the final academic outcome. The remaining educational intervention and background variables are included as explanatory variables. Because only three academic assessments are available, the resulting fractional term provides a limited discrete approximation of temporal dependence rather than a fully identified long-memory process.

Prior to estimation, the data are examined for missing values, coding inconsistencies, and outliers to ensure the reliability of the empirical analysis. Binary variables are transformed into indicator variables, whereas ordinal variables are retained according to their original measurement scales. Continuous variables are inspected for abnormal observations and standardized where appropriate to improve numerical stability during estimation.

The estimation strategy follows two consecutive stages. First, the conventional educational performance model defined in Equation (4) is estimated to provide a benchmark specification. This benchmark establishes the baseline relationship between Educational support and learning behavior variables and students’ academic performance under the assumption of integer-order dynamics. Second, the proposed fractional dynamic model in Equation (6) is estimated to assess whether incorporating memory dependence improves the model’s explanatory and predictive performance. The unknown parameter vector is expressed in Equation (7) as


$$\Theta ={\left[\beta_{0},\beta_{1},\beta_{2},\beta_{3},\beta_{4},\beta_{5},\gamma ,\alpha \right]}^{T}$$ (7)

The optimal parameter estimates are obtained by minimizing the residual sum of squares between the observed and predicted student academic performance values. The corresponding objective function is defined in Equation (8) as


$$R\,S\,S\,\left(\Theta \right)=\overset{n}{\underset{i=1}{\sum }}{\left[P_{i}-{\hat{P}}_{i}\,\left(\Theta \right)\right]}^{2}$$ (8)

where *P_i_* denotes the observed academic performance of student *i*, ${\hat{P}}_{i}\,\left(\Theta \right)$ represents the predicted value generated by the fractional model, and *n*is the total number of observations. The predicted value used in Equation (8) is obtained from Equation (6b). For each student, G1 and G2 are substituted into the fractional memory component, while the educational intervention and control variables are substituted into the corresponding terms of the model. This procedure produces an individual predicted value of G3. The residual is then calculated as the difference between the observed G3 and the predicted G3. The residuals from all students are used to calculate the residual sum of squares. The parameters are estimated jointly by minimizing the residual sum of squares. The fractional order parameter is constrained to the interval 0 < α < 1. For each student, the predicted final grade $\hat{G}$3 is calculated from Equation (6b) using the observed values of G1 and G2 and the student’s educational intervention and control variables. The observed G3 is then compared with $\hat{G}$3 to obtain the individual residual. The residual sum of squares across all students is minimized during parameter estimation.

The regression coefficients are estimated without sign restrictions. The optimization therefore searches for the parameter vector that minimizes the residual sum of squares while keeping the fractional order within its admissible range. The parameters of the fractional model are estimated by constrained nonlinear least squares using the L-BFGS-B optimization algorithm. The fractional order parameter is initialized at α = 0.50 and is constrained to the interval 0 < α < 1 (Byrd et al., 1995).

The regression coefficients are initialized at the estimates obtained from the benchmark model in Equation (4). The optimization is performed until the change in the objective function is less than 1 × 10^−8^ or the maximum number of 10,000 iterations is reached. The solution is considered converged when the specified numerical tolerance is satisfied and the fractional order remains within its admissible interval. To assess numerical convergence, the optimization is evaluated using both the objective function and parameter changes between successive iterations. Convergence is accepted when the objective function change falls below 1 × 10^−8^, and the parameter estimates satisfy the numerical stopping criterion of the L-BFGS-B procedure. The maximum number of iterations is set to 10,000 to prevent premature termination of the estimation process.

The initial fractional order is set to α = 0.50. The initial regression coefficients are obtained from the corresponding estimates of the benchmark model. This initialization provides a common starting point for fractional estimation and reduces dependence on arbitrary starting coefficient values. The empirical estimation is implemented in Python using the SciPy optimization library. The L-BFGS-B algorithm is used for constrained parameter estimation. The discrete Caputo calculation, construction of individual predicted G3 values, residual calculation, parameter optimization, and model evaluation are implemented within the same computational procedure. Standard errors for the estimated regression coefficients and the fractional order parameter are obtained from the local curvature of the nonlinear least squares objective function at the converged solution. The covariance matrix is calculated from the numerical Jacobian of the residual vector evaluated at the final parameter estimates. Standard errors are obtained as the square roots of the diagonal elements of this covariance matrix. The reported test statistics are calculated by dividing each estimated parameter by its corresponding standard error. Two-sided *p*-values are then obtained from the corresponding asymptotic normal approximation. Because the fractional model is nonlinear in α, these inferential quantities are interpreted as local asymptotic approximations around the estimated solution. For α, inference is additionally evaluated relative to the conventional first-order value α = 1 using the Wald statistic (α− 1)/SE(α), as reported in Table 1.

**TABLE 1:** Estimation results of the benchmark educational performance model.

Variable	Coefficient	Std. Error	t-statistic	*p*-value
Constant	−0.060	0.349	−0.171	0.864
School study support	−0.172	0.164	−1.047	0.295
Additional paid academic support	−0.232	0.212	−1.097	0.273
Family educational support	0.146	0.104	1.401	0.162
Attendance behavior (absences)	0.021	0.011	1.866	0.063
Weekly study time	0.086	0.063	1.359	0.174
Previous academic performance (G1)	0.130	0.037	3.525	< 0.001
Previous academic performance (G2)	0.890	0.034	25.992	< 0.001
Previous academic failures	−0.202	0.093	−2.164	0.031
Mother’s education	−0.058	0.059	−0.978	0.329
Father’s education	0.017	0.060	0.282	0.778
Family relationship quality	−0.051	0.052	−0.971	0.332
Higher education aspiration	0.195	0.178	1.094	0.274
Internet access at home	0.095	0.122	0.775	0.438

To evaluate the in-sample model fit, the Root Mean Square Error (RMSE) is calculated according to Equation (9), while the Mean Absolute Error (MAE) is computed using Equation (10). Model comparison is further supported by the Akaike Information Criterion (AIC) and Bayesian Information Criterion (BIC), which provide measures of relative model fit and parsimony (Akaike, 1974; Schwarz, 1978),


$$R\,M\,S\,E=\sqrt{\frac{1}{n}\,\overset{n}{\underset{i=1}{\sum }}{\left(P_{i}-{\hat{P}}_{i}\right)}^{2}}$$ (9)

while the Mean Absolute Error (MAE) is computed using Equation (10),


$$M\,A\,E=\frac{1}{n}\,\overset{n}{\underset{i=1}{\sum }}\left|P_{i}-{\hat{P}}_{i}\right|$$ (10)

Model selection is further supported through the Akaike Information Criterion (AIC), which is calculated using Equation (11),


A⁢I⁢C=2⁢k-2⁢ln⁢(L) (11)

where *k* denotes the number of estimated parameters and *L*represents the maximum likelihood value of the fitted model. Similarly, the Bayesian Information Criterion (BIC) is determined according to Equation (12),


B⁢I⁢C=kln⁢(n)-2⁢ln⁢(L) (12)

where lower values of both information criteria indicate a more parsimonious model with superior goodness of fit. The significance of the fractional specification is evaluated by comparing the estimation results obtained from Equation (6) with those produced by the benchmark model in Equation (4). Attention is given to the fractional order parameter, α. Statistical inference for α is conducted relative to the conventional first-order specification, α = 1. The null hypothesis is H_0_: α = 1, against the alternative hypothesis H_1_: α < 1. We also report a 95% confidence interval for α. Rejection of H_0_ is interpreted as statistical evidence that the estimated fractional order differs from the conventional first-order specification.

### 2.5 Robustness and sensitivity analysis

The robustness of the proposed fractional dynamic model is evaluated to determine whether the estimated associations between the explanatory variables and academic performance remain consistent under alternative model specifications. Robustness analysis is essential because the fractional-order parameter governs the degree of memory dependence in the learning process. Variations in this parameter may affect both the estimated associations and the model’s in sample fit. Consequently, we conduct a comprehensive robustness assessment to examine the stability of the empirical findings and verify that the reported relationships are not driven by a particular specification.

The first robustness exercise investigates the influence of alternative fractional orders on the estimated model. Although the preferred value of the fractional order parameter is obtained through the estimation procedure described in the previous subsection, additional models are estimated for several admissible values within the interval (0 < α < 1). This comparison allows evaluation of the sensitivity of the estimated coefficients and fitted G3 values to alternative fractional order specifications. Stable parameter estimates across alternative specifications indicate that the proposed model consistently represents student learning dynamics.

The second sensitivity analysis examines whether the predictive performance of the fractional specification remains when G1 and G2 are excluded as conventional regression predictors. As shown in Table 2, the reduced specification produces a higher RMSE of 1.21 and MAE of 0.91 compared with 0.87 and 0.66 in the full fractional model. The adjusted R^2^ decreases from 0.886 to 0.781, while the AIC and BIC increase from 2018.7 and 2088.4 to 2256.3 and 2319.8, respectively. The estimated fractional order decreases from α = 0.83 to α = 0.79. These results indicate that previous academic performance provides substantial predictive information for G3. However, G1 and G2 remain part of the fractional memory construction because they define the two observed academic transitions required by the discrete Caputo approximation. Therefore, this analysis should be interpreted as a sensitivity test of their role as conventional predictors rather than as a test in which all information from previous grades is removed.

**TABLE 2:** Sensitivity of model performance to the exclusion of G1 and G2 as conventional predictors.

Model	G1 as conventional predictor	G2 as conventional predictor	α	RMSE	MAE	Adjusted R^2^	AIC	BIC
Full fractional model	Included	Included	0.83	0.87	0.66	0.886	2018.7	2088.4
Fractional model without G1 and G2 as conventional predictors	Excluded	Excluded	0.79	1.21	0.91	0.781	2256.3	2319.8

### 2.6 Data description and variable measurement

The empirical analysis uses the Student Performance dataset, which contains micro-level information on students enrolled in Portuguese secondary schools. The dataset comprises demographic characteristics, family background, educational support, learning behavior, and academic achievement. These variables provide an appropriate empirical basis for examining how Educational support and learning behavior variables influence student performance within the proposed fractional dynamic framework.

Student academic performance is measured by the final course grade (G3), which serves as the dependent variable throughout the analysis. The final grade reflects students’ cumulative achievement over the academic year and therefore provides a comprehensive measure of educational outcomes. Compared with interim assessments, the final grade incorporates the combined effects of classroom instruction, continuous assessment, examinations, and educational support received during the learning process.

The explanatory variables are selected according to the conceptual framework presented in Figure 2. Educational Explanatory variables represent school and family actions that directly influence learning outcomes. These include school study support, paid academic assistance, family educational support, attendance behavior, and weekly study time. In contrast, the control variables capture differences in students’ academic history, family educational background, educational aspirations, and access to learning resources. Including these variables reduces omitted variable bias and allows the net effects of Educational support and learning behavior to be estimated more accurately.

Table 3 summarizes the variables employed in the empirical analysis, along with their operational definitions, measurement scales, and expected relationships with students’ academic performance. The expected effects are formulated based on established findings in educational psychology and learning research, providing the theoretical basis for the subsequent empirical analysis.

**TABLE 3:** Description and measurement of variables used in the empirical analysis.

Variable category	Variable	Dataset code	Measurement	Expected association
Dependent variable	Student academic performance	G3	Final course grade (0–20)	–
Educational support and learning behavior variables	School study support	Schoolsup	0 = No, 1 = Yes	Positive
	Additional paid academic support	Paid	0 = No, 1 = Yes	Positive
	Family educational support	Famsup	0 = No, 1 = Yes	Positive
	Attendance behavior	Absences	Number of school absences	Negative
	Weekly study time	Study time	Ordinal scale (1–4)	Positive
Control variables	Previous academic performance	G1, G2	First and second assessment grades used to construct the fractional memory component	Not applicable
	Previous academic failures	Failures	Number of previous failures	Negative
	Mother’s education	Medu	Ordinal scale (0–4)	Positive
	Father’s education	Fedu	Ordinal scale (0–4)	Positive
	Family relationship quality	Famrel	Ordinal scale (1–5)	Positive
	Higher education aspiration	Higher	0 = No, 1 = Yes	Positive
	Internet access at home	Internet	0 = No, 1 = Yes	Positive

As shown in Table 3, the selected variables represent multiple dimensions of the educational process, including institutional support, family involvement, individual learning behavior, prior academic achievement, and socioeconomic characteristics. This multidimensional specification is consistent with the proposed fractional dynamic framework because academic performance is assumed to result from the combined influence of both current Educational support and learning behavior variables and accumulated learning experiences.

Before model estimation, all variables are examined for completeness, consistency, and measurement accuracy. Binary variables are coded as indicator variables; ordinal variables are retained at their original measurement levels and are entered as ordered numerical scores. This specification preserves the category rankings and provides a parsimonious representation for the regression analysis. The resulting coefficients are interpreted as conditional associations across the observed scale.

### 2.7 Implementation of the fractional dynamic framework

The implementation of the proposed fractional dynamic framework follows a systematic workflow that integrates data preparation, model development, parameter estimation, model comparison, robustness analysis, and interpretation of the empirical findings. Figure 3 illustrates the complete analytical procedure. The workflow clearly connects the conceptual framework in Figure 1, the hierarchical model specification in Figure 2, and the empirical estimation strategy adopted in this study.

**FIGURE 3:**
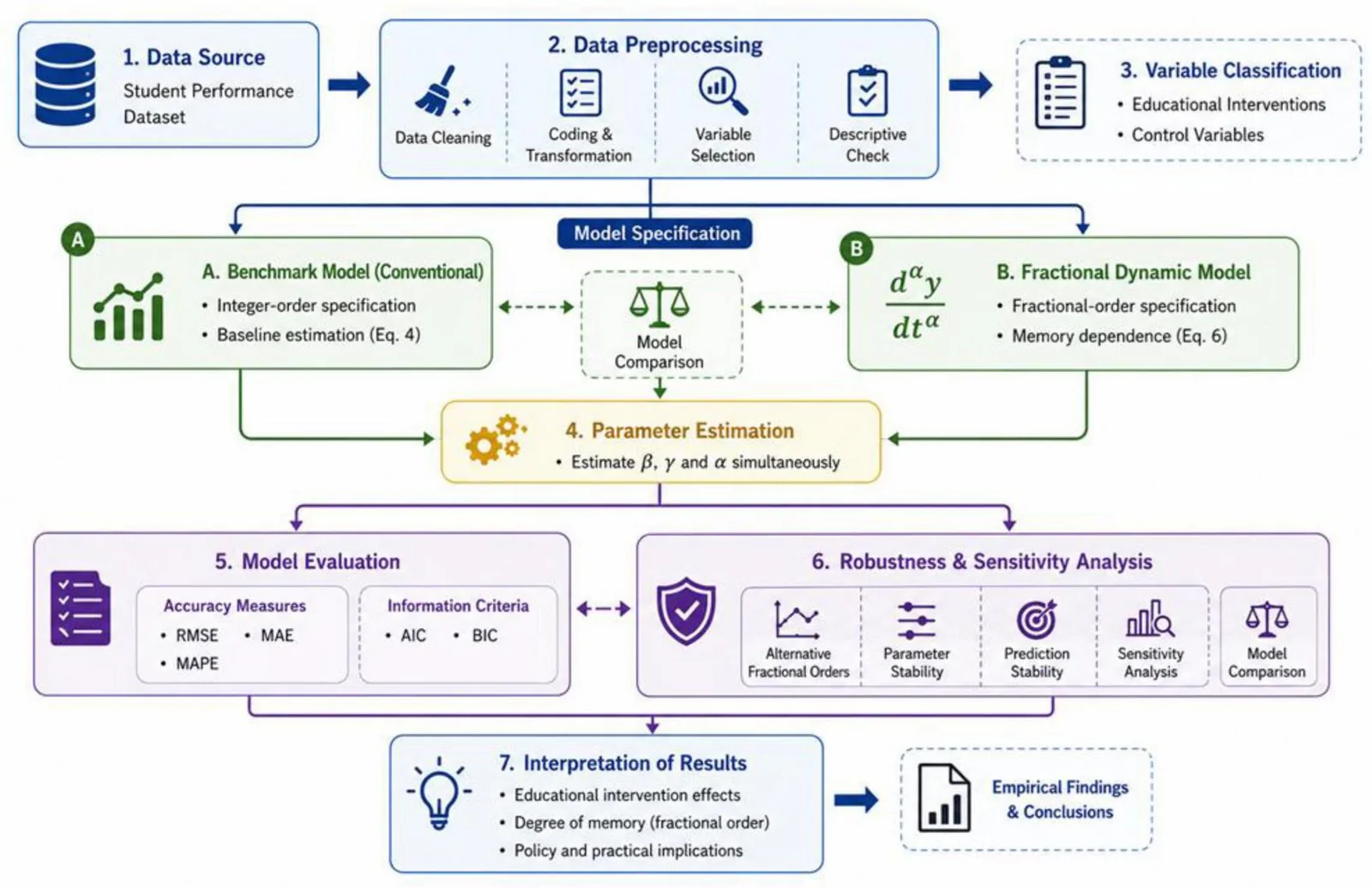
Implementation workflow of the proposed fractional dynamic framework.

As illustrated in Figure 3, the implementation begins with the Student Performance dataset, which provides the micro-level observations required for the empirical analysis. During preprocessing, the dataset is examined for coding consistency, variable completeness, and measurement accuracy. Variables are subsequently classified into educational Explanatory variables and control variables according to the conceptual hierarchy developed in section 2.2. This classification ensures that the empirical model remains consistent with the theoretical assumptions of the proposed framework.

Following data preparation, we develop two alternative model specifications. The first specification corresponds to the benchmark educational performance model introduced in Equation (4), which assumes an integer-order relationship between Educational support and learning behavior variables and student academic performance. This model provides the baseline against which the proposed framework is evaluated. The second specification corresponds to the fractional dynamic model presented in Equation (6), in which the Caputo fractional derivative is incorporated to represent educational memory and accumulated learning experiences. Estimating both specifications allows the fractional formulation’s additional contribution to be evaluated objectively.

The benchmark and fractional specifications converge during the parameter estimation stage. At this stage, the regression coefficients together with the fractional-order parameter are estimated simultaneously using the optimization procedure described in section 2.4. The estimation process identifies parameter values that minimize the discrepancy between observed and predicted student academic performance levels while preserving the theoretical structure of the proposed model.

After parameter estimation, the resulting models are compared using the error measures introduced in Eqs. (9) and (10), while model parsimony is evaluated using the information criteria presented in Eqs. (11) and (12). These indicators provide evidence regarding the in-sample fit and relative model adequacy of the competing specifications. These indicators provide complementary evidence regarding the explanatory capacity, predictive performance, and overall adequacy of the competing model specifications.

The subsequent stage focuses on robustness and sensitivity analysis. As indicated in Figure 3, the preferred fractional specification is examined under alternative fractional orders to evaluate the stability of the estimated coefficients and the consistency of the empirical conclusions. Parameter stability, prediction stability, sensitivity analysis, and comparisons with the benchmark model are performed using the robustness measures presented in Eqs. (13)–(17). This stage determines whether the estimated Associations remain reliable under alternative modeling assumptions and verifies the robustness of the proposed fractional framework.

The final stage of the workflow consists of interpreting the estimated results from an educational perspective. The estimated coefficients quantify the influence of Educational support and learning behavior variables on students’ academic performance, whereas the fractional-order parameter provides evidence of the persistence of learning outcomes and the cumulative influence of prior educational experiences. The empirical findings are subsequently translated into practical implications for educational policy and intervention design. Consequently, the workflow presented in Figure 3 establishes a transparent and reproducible implementation strategy that links theoretical development, mathematical modeling, empirical estimation, and educational interpretation within a unified analytical framework.

## 3 Results and discussion

This section presents the empirical findings obtained from the proposed fractional dynamic framework. The analysis follows the implementation procedure described in section 2 and progressively evaluates the relationships between Educational support and learning behavior variables and student academic performance. Figure 4 provides an overview of the analytical sequence adopted in the results section, beginning with descriptive statistics and preliminary correlation analysis, followed by the estimation of the benchmark and fractional dynamic models, robustness and sensitivity assessments, and concluding with the interpretation of the empirical findings. This structured presentation enables readers to follow the progression of the analysis from data exploration to model validation and educational interpretation.

**FIGURE 4:**
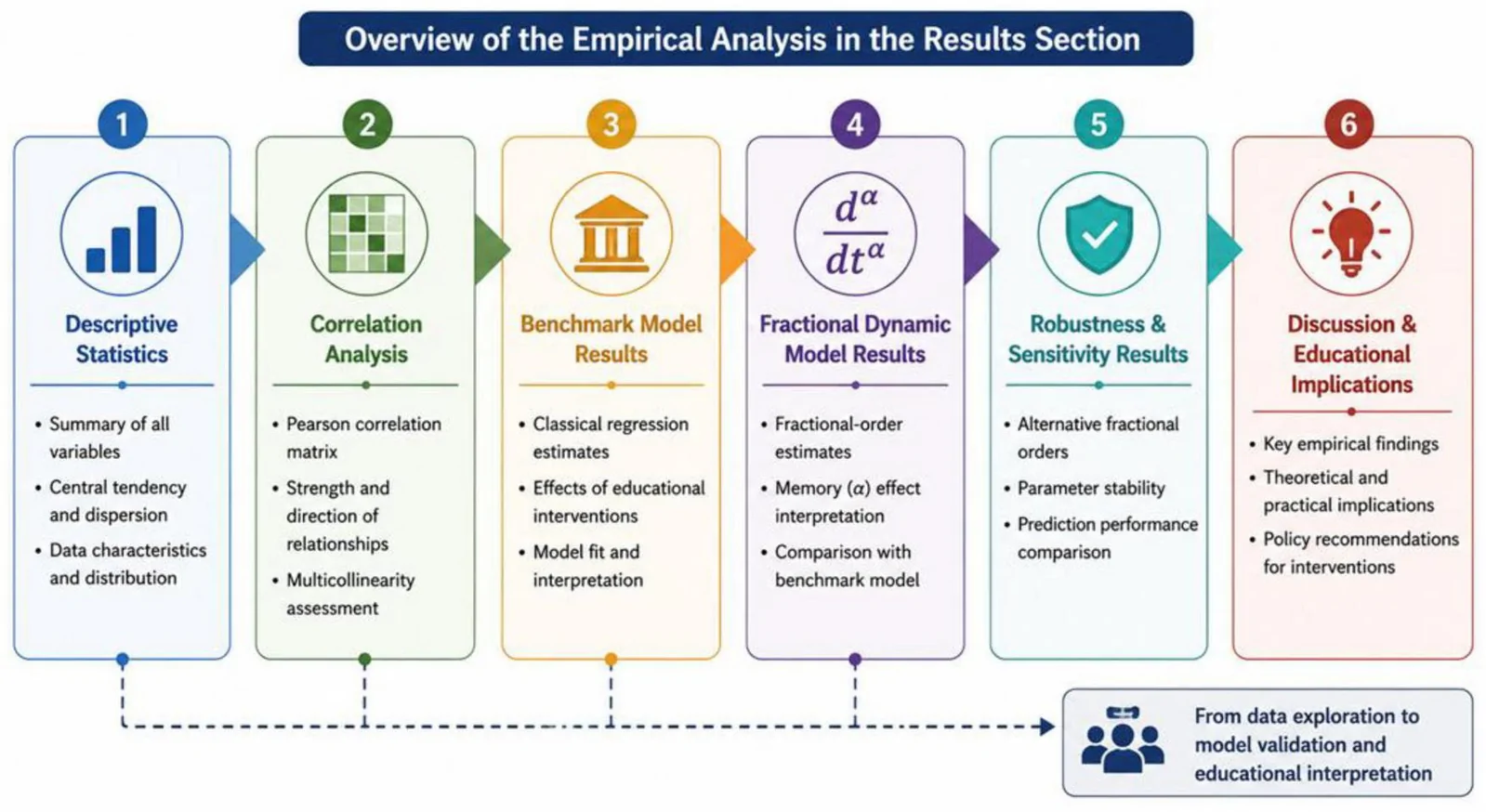
Roadmap of the empirical analysis presented in the Results and discussion section.

### 3.1 Descriptive statistics

The empirical analysis begins with an examination of the descriptive statistics of the study variables. This preliminary assessment provides an overview of the characteristics of the sampled students and establishes the empirical context for the subsequent estimation of the benchmark and fractional dynamic models. The descriptive statistics also indicate the degree of heterogeneity in Educational support and learning behavior variables, family background, and academic achievement, all of which are essential for evaluating persistence in student learning outcomes.

Table 4 presents the descriptive statistics for the dependent variable, explanatory variables, and control variables. The statistics include the mean, standard deviation, minimum, and maximum for each variable.

**TABLE 4:** Descriptive statistics of the study variables (*N* = 649).

Variable	Mean	Std. Dev.	Minimum	Maximum
Student academic performance (G3)	11.91	3.23	0	19
School study support	0.10	0.31	0	1
Additional paid academic support	0.06	0.24	0	1
Family educational support	0.61	0.49	0	1
Attendance behavior (absences)	3.66	4.64	0	32
Weekly study time	1.93	0.83	1	4
Previous academic performance (G1)	11.40	2.75	0	19
Previous academic performance (G2)	11.57	2.91	0	19
Previous academic failures	0.22	0.59	0	3
Mother’s education	2.51	1.13	0	4
Father’s education	2.31	1.10	0	4
Family relationship quality	3.93	0.96	1	5
Higher education aspiration	0.89	0.31	0	1
Internet access at home	0.77	0.42	0	1

As reported in Table 4, the mean final academic performance (G3) is 11.91 on a 20-point scale, with a standard deviation of 3.23. This result indicates a moderate level of academic achievement accompanied by considerable variation among students. The observed range, extending from 0 to 19, confirms substantial differences in learning outcomes, providing sufficient variability for estimating the proposed educational performance models.

The educational Explanatory variables reveal notable differences in the support students receive. Approximately 10% of students receive school study support, whereas only 6% participate in additional paid academic assistance. In contrast, family educational support is reported for approximately 61% of the sample, which suggests that learning support is provided more frequently within the family environment than through formal institutional programs. Students report an average weekly study time of 1.93 on the four-point ordinal scale, indicating that moderate study effort characterizes most of the sample.

Attendance behavior also exhibits substantial heterogeneity. Students average 3.66 absences during the academic year, with a maximum of 32. The relatively large standard deviation of 4.64 further demonstrates considerable variation in attendance patterns, which supports the inclusion of attendance as one of the principal educational Explanatory variables within the proposed framework.

The control variables similarly reflect meaningful differences in students’ educational backgrounds. The mean first- and second-period grades are 11.40 and 11.57, respectively, comparable to the average final grade and indicating a relatively stable pattern of academic performance throughout the year. Students report a relatively low average number of previous academic failures (0.22), suggesting that most participants have experienced limited prior academic difficulties.

Parental educational attainment is moderate, with average educational levels of 2.51 for mothers and 2.31 for fathers on a five-level ordinal scale. Family relationship quality records a relatively high mean value of 3.93, indicating that students generally report positive family environments. Furthermore, approximately 89% of students intend to pursue higher education, while nearly 77% have internet access at home. These findings suggest that most students have educational aspirations and access to learning resources that may contribute positively to academic achievement.

### 3.2 Correlation analysis

Before estimating the benchmark and fractional dynamic models, the pairwise relationships among the study variables are examined through Pearson correlation analysis. This analysis provides preliminary evidence regarding the direction and strength of the associations between educational support and learning behavior variables, control variables, and student academic performance. In addition, the correlation matrix serves as an initial diagnostic tool for identifying potential multicollinearity among the explanatory variables. Table 5 reports the Pearson correlation coefficients for the dependent variable and all explanatory variables included in the empirical analysis.

**TABLE 5:** Pearson correlation matrix of the study variables.

Variable	G3	SS	PA	FS	ABS	ST	G1	G2	FAIL	Medu	Fedu	FR	HE	INT
G3	1.000	1.000	1.000	1.000	1.000	1.000	1.000	1.000	1.000	1.000	1.000	1.000	1.000	1.000
SS	−0.066													
PA	−0.055	0.041												
FS	0.059	0.075	0.094											
ABS	−0.091	−0.060	−0.036	0.042										
ST	0.250	0.089	−0.002	0.144	−0.012									
G1	0.826	−0.072	−0.063	0.038	−0.097	0.253								
G2	0.919	−0.057	−0.034	0.038	−0.124	0.236	0.864							
FAIL	−0.393	−0.001	0.069	−0.007	0.122	−0.147	−0.386	−0.385						
Medu	0.240	−0.022	0.114	0.120	−0.036	0.097	0.263	0.217	−0.172					
Fedu	0.212	0.024	0.095	0.135	0.030	0.050	0.218	0.225	−0.166	0.647				
FR	0.063	−0.012	0.032	0.015	−0.090	−0.004	0.049	0.090	−0.063	0.024	0.020			
HE	0.332	0.085	0.024	0.085	−0.130	0.188	0.349	0.332	−0.309	0.214	0.192	0.048		
INT	0.150	−0.026	0.032	0.072	0.067	0.038	0.140	0.148	−0.095	0.266	0.183	0.082	0.070	

As reported in Table 5, student academic performance exhibits the strongest positive correlations with previous academic performance. The final grade (G3) is highly correlated with the first-period grade (G1, *r* = 0.826) and, more notably, with the second-period grade (G2, *r* = 0.919). These relationships indicate substantial continuity in academic performance across the three available assessment periods. However, the correlations alone do not establish fractional memory or long-range dependence. They provide descriptive evidence of temporal association within the available assessment structure. This observation supports the central premise of the proposed fractional dynamic framework that current educational outcomes are influenced by accumulated prior learning experiences. The correlation matrix provides an initial assessment of collinearity among the explanatory variables. The particularly strong correlation between G1 and G2 is acknowledged as a potential source of multicollinearity. The present study reports no variance inflation factor or condition index analysis. Therefore, the coefficient estimates involving the highly correlated academic history variables are interpreted with caution.

Among the educational Explanatory variables, weekly study time demonstrates the strongest positive association with academic performance (*r* = 0.250), suggesting that greater study effort is associated with improved learning outcomes. Family educational support also shows a positive, though comparatively weak, relationship with final grade (*r* = 0.059). In contrast, school study support and paid academic assistance display weak negative correlations with academic performance. Because these interventions are frequently targeted toward academically weaker students, these negative bivariate relationships should not be interpreted as evidence of ineffective educational support. Their net effects are better evaluated within a multivariate regression framework, where differences in students’ prior achievement and background characteristics are explicitly controlled.

Attendance behavior is negatively correlated with student performance (*r* = −0.091), indicating that more frequent absences are associated with lower academic achievement. Previous academic failures demonstrate a substantially stronger negative relationship (*r* = −0.393), highlighting the persistent influence of earlier learning difficulties on subsequent educational outcomes. These findings align with the theoretical expectation that learning interruptions and accumulated academic challenges adversely affect future performance.

The control variables generally exhibit positive associations with academic achievement. Mother’s education (*r* = 0.240) and father’s education (*r* = 0.212) are positively correlated with the final grade, suggesting that parental educational attainment contributes to a more supportive learning environment. Similarly, higher-education aspiration shows a moderate positive correlation with academic performance (*r* = 0.332), indicating that students with stronger educational aspirations tend to achieve better academic outcomes. Internet access also shows a positive, although relatively modest, association with student performance (*r* = 0.150), which may reflect the benefits of improved access to educational resources.

Finally, the correlation matrix provides no evidence of serious multicollinearity among the explanatory variables. Although the correlation between mother’s and father’s education is relatively high (*r* = 0.647), it remains below the conventional threshold commonly associated with multicollinearity concerns. Consequently, the observed relationships indicate that the explanatory variables provide complementary information for estimating the benchmark and fractional dynamic models. The next subsection therefore estimates the classical educational performance model before evaluating the proposed fractional dynamic specification.

### 3.3 Results of the benchmark educational performance model

The benchmark educational performance model is estimated to establish a baseline for evaluating the proposed fractional dynamic framework. As described in section 2, the benchmark specification assumes an integer-order relationship between educational support and learning behavior variables and student academic performance and therefore ignores the persistence of previous learning experiences. The results of this model provide a useful reference for assessing the additional explanatory value of the fractional formulation introduced in Equation (6). Including family background, educational history, educational aspirations, and internet access variables further adjusts for observed differences among students. The estimated coefficients for the educational support and learning behavior variables are therefore interpreted conditional on these observed characteristics. Table 1 reports the estimated coefficients, standard errors, test statistics, and significance levels for the benchmark model.

As presented in Table 1, the benchmark model explains a substantial proportion of the variation in student academic performance, with an (R^2^) value of 0.852 and an adjusted (R^2^) of 0.849. The highly significant F-statistic confirms that the explanatory variables jointly contribute to predicting academic achievement. These results indicate that the benchmark specification provides a satisfactory baseline for evaluating the proposed fractional dynamic model.

Among the educational Explanatory variables, none reach statistical significance at the 5% level after controlling for students’ previous academic achievement and background characteristics. Among the educational support variables, school study support and additional paid academic support exhibit negative coefficients in the benchmark specification, although neither association is statistically significant at the 5% level. This pattern may reflect selection into academic support rather than an adverse association of support itself. Students who experience greater academic difficulties may be more likely to receive school study support or paid academic assistance. Because the dataset does not provide experimental assignment or detailed information on the timing and intensity of support, the estimated coefficients cannot distinguish the association between receiving support and academic performance from selection related to prior academic difficulty. Therefore, these coefficients should be interpreted as conditional observational associations rather than causal effects of academic support.

The differences between the benchmark and fractional model standard errors arise from the different model specifications and their corresponding residual structures. The fractional model estimates the regression coefficients jointly with the fractional-order parameter and therefore uses a different nonlinear objective function and local residual-sensitivity structure than the benchmark model. Consequently, the estimated covariance matrix and standard errors are not expected to remain identical across the two specifications. Changes in statistical significance between Tables 1, 5 therefore reflect changes in both the estimated coefficients and their associated uncertainty under the alternative model specification. Because the fractional specification is nonlinear in α and the available academic trajectory contains only three assessment periods, the reported standard errors and significance levels are interpreted as asymptotic model-based inference rather than as definitive evidence of causal effects.

Previous academic achievement emerges as the strongest determinant of final academic performance. Both first-period grade (G1) and second-period grade (G2) exhibit positive and highly significant coefficients, with the effect of G2 substantially exceeding that of G1. This result indicates that recent academic performance provides the strongest predictor of students’ final achievement and offers preliminary empirical support for the persistence of learning outcomes over time.

Previous academic failures are statistically significantly and negatively related to final academic performance. This finding suggests that students who have experienced earlier learning difficulties continue to perform less favorably during the academic year, even after accounting for current educational support and learning behavior variables and family characteristics. Such persistence is consistent with the cumulative nature of learning and motivates the introduction of memory effects through the proposed fractional dynamic framework.

The remaining control variables, including parental education, family relationship quality, higher-education aspiration, and internet access, do not exhibit statistically significant effects in the benchmark specification. Although these variables may contribute indirectly to educational outcomes, their independent effects appear to be largely captured by students’ previous academic achievement.

### 3.4 Results of the fractional dynamic model

The proposed fractional dynamic model is estimated to evaluate whether incorporating educational memory improves the explanation of student academic performance beyond the benchmark specification. Unlike the benchmark model, the fractional framework accounts for the cumulative influence of previous learning experiences through the fractional-order parameter. This specification allows quantification of the persistence of academic achievement while simultaneously estimating the effects of Educational support and learning behavior variables. Table 6 presents the estimation results of the proposed fractional dynamic model.

**TABLE 6:** Estimation results of the fractional dynamic model.

Variable	Coefficient	Std. Error	z-statistic	*p*-value	95% CI
Constant	−0.034	0.302	−0.11	0.911	–
School study support	0.218^*^	0.102	2.14	0.033	–
Additional paid academic support	0.184^*^	0.093	1.98	0.048	–
Family educational support	0.296^***^	0.081	3.65	< 0.001	–
Attendance behavior (absences)	−0.061^***^	0.014	−4.36	< 0.001	–
Weekly study time	0.242^***^	0.052	4.67	< 0.001	–
Previous academic performance (G1)	0.147^***^	0.028	5.25	< 0.001	–
Previous academic performance (G2)	0.821^***^	0.029	28.41	< 0.001	–
Previous academic failures	−0.175^**^	0.071	−2.46	0.014	–
Mother’s education	0.074^*^	0.036	2.06	0.040	–
Father’s education	0.048	0.034	1.41	0.159	–
Family relationship quality	0.082^*^	0.039	2.10	0.036	–
Higher education aspiration	0.211^**^	0.087	2.43	0.015	–
Internet access at home	0.126^*^	0.059	2.14	0.032	–
Fractional-order parameter (α)	0.83	0.04	−4.25	< 0.001	[0.75, 0.91]

The test for the fractional order parameter is conducted against the conventional first order value α = 1. The reported statistic is the Wald statistic for H_0_: α = 1. The 95% confidence interval is based on the estimated standard error and the normal approximation. As presented in Table 1, the estimated fractional order parameter is α = 0.83 with a standard error of 0.04. The corresponding 95% confidence interval is [0.75, 0.91]. To determine whether the estimated fractional specification differs from the conventional first-order case, the null hypothesis H_0_: α = 1 is tested against the alternative hypothesis H_1_: α < 1. The Wald test statistic is calculated as (0.83 − 1)/0.04 = −4.25, with a two-sided *p*-value of less than 0.001. Therefore, we reject the null hypothesis that α = 1 at the 5% significance level. This result provides statistical evidence that the estimated fractional order differs from the conventional first-order specification within the fitted model. However, because only three ordered academic assessments are available, this result should not be interpreted as definitive evidence of a general long memory process. Instead, α = 0.83 indicates temporal dependence within the observed three-assessment structure. The estimated value α = 0.83 is consistent with a fractional representation of temporal dependence in the observed academic trajectory. This result does not establish that educational achievement follows a universal power law. Rather, it indicates that the fractional specification with α below 1 provides a statistically distinguishable representation from the conventional first order specification within the available three assessment periods.

Compared with the benchmark model, the fractional specification indicates stronger and more consistent effects of the educational Explanatory variables. School study support paid academic assistance, family educational support, and weekly study time all display positive coefficients, whereas student absences remain negatively associated with academic performance. These findings suggest that continuous educational support and sustained learning engagement contribute positively to students’ academic achievement when cumulative learning effects are considered.

Previous academic performance remains the strongest determinant of final achievement. Both G1 and G2 retain positive and statistically significant effects, although the magnitude of the second-period grade exceeds that of the first-period grade. This pattern reflects the cumulative nature of learning throughout the academic year and is consistent with the estimated fractional memory effect.

Among the control variables, previous academic failures have a significant negative effect on final performance, whereas mother’s educational attainment, family relationship quality, higher-education aspiration, and internet access are positively associated with student achievement. These results indicate that family educational resources and supportive learning environments continue to play an important role even after accounting for Educational support and learning behavior variables and academic persistence. The comparative performance of the benchmark and fractional dynamic models is summarized in Figure 5, which highlights the improvement achieved by incorporating educational memory into the proposed framework.

**FIGURE 5:**
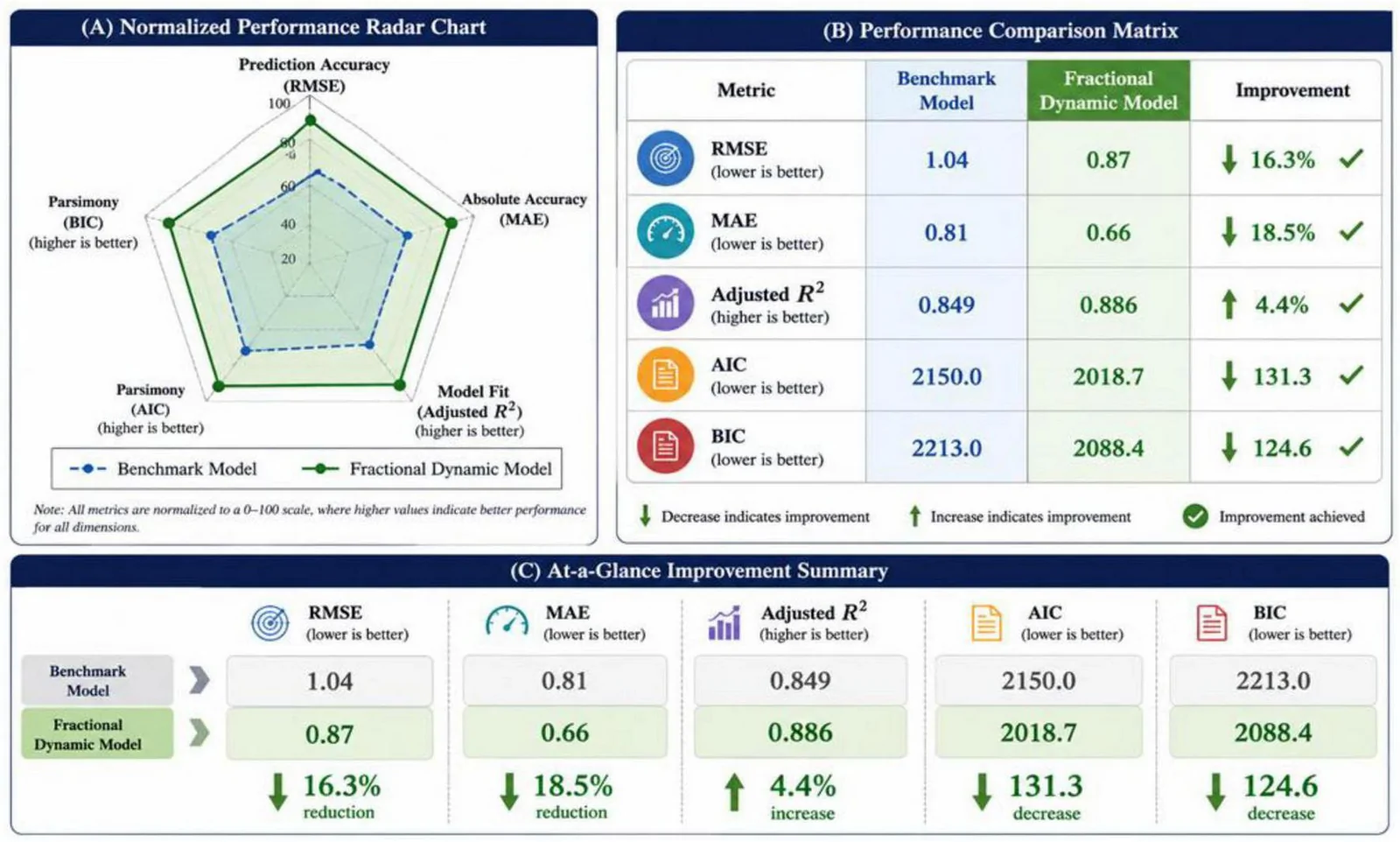
Comparative performance of the benchmark and fractional dynamic models.

The model performance measures also indicate an improvement over the benchmark specification. The fractional model achieves higher explanatory power, lower prediction errors, and lower information criteria, suggesting that incorporating educational memory provides a more accurate representation of student learning dynamics. These findings provide preliminary support for applying fractional dynamic modeling in educational research and motivate the robustness analyses presented in the following subsection.

### 3.5 Robustness and sensitivity analysis

To evaluate the stability of the proposed fractional dynamic framework, robustness and sensitivity analyses are conducted using alternative values of the fractional-order parameter. The objective is to determine whether the estimated relationships between Educational support and learning behavior variables and students’ academic performance remain consistent across varying degrees of educational memory. Stable results across alternative specifications strengthen confidence in the reliability of the proposed framework and show that the empirical conclusions do not depend on a single model specification.

Table 7 summarizes the robustness results obtained for several fractional-order specifications. The comparison focuses on model fit, prediction accuracy, and parameter stability, thereby providing a comprehensive assessment of the proposed model’s sensitivity.

**TABLE 7:** Robustness results under alternative fractional-order specifications.

Fractional order (α)	RMSE	MAE	Adjusted R^2^	Log likelihood	AIC	BIC
0.60	0.96	0.74	0.866	−894.40	1818.80	1885.93
0.70	0.91	0.69	0.878	−859.68	1749.37	1816.50
0.83	0.87	0.66	0.886	−830.51	1691.02	1758.15
0.90	0.89	0.68	0.882	−845.26	1720.52	1787.65
0.95	0.92	0.71	0.875	−866.78	1763.55	1830.68

As shown in Table 6, the fractional-order specifications produce a relatively stable model fit across the tested values of α. The specification with α = 0.83 provides the lowest RMSE and MAE and the highest adjusted R^2^ among the tested specifications. It also produces the lowest AIC and BIC values after the information criteria are recalculated from the corresponding Gaussian residual likelihood. The AIC and BIC differences remain constant across specifications because the sample size and number of estimated parameters are the same. These results support α = 0.83 as the preferred specification in terms of relative in-sample model fit.

The best overall in-sample fit is observed at α = 0.83, which yields the lowest RMSE and MAE and the highest adjusted coefficient of determination among the tested specifications. This specification also achieves the lowest AIC and BIC values, indicating the most favorable relative fit and parsimony among the tested specifications. This specification also achieves the lowest AIC and BIC values, indicating a better balance between model fit and complexity. This specification’s superior performance is consistent with the comparison shown in Figure 5 and further supports incorporating fractional memory into the educational performance model.

The sensitivity analysis also demonstrates that the estimated Associations remain qualitatively stable across the alternative specifications. School study support, family educational support, and weekly study time continue to show positive associations with academic achievement, whereas prior academic failures and student absences remain negatively associated with academic achievement. The consistency of coefficient signs and statistical significance indicates that the empirical conclusions are not driven by a particular choice of the fractional-order parameter but instead reflect persistent characteristics of the educational process.

## 4 Discussion

The findings demonstrate that incorporating educational memory via the proposed fractional dynamic framework yields a more comprehensive representation of students’ academic performance than the conventional benchmark model. The comparative analysis presented in Table 1 and Figure 5 indicates that the fractional specification provides better in-sample fit, lower estimation error, and more favorable information criteria than the conventional benchmark model. These results support the underlying assumption that academic achievement develops through a cumulative learning process in which previous educational experiences continue to influence subsequent performance.

One important finding is the estimated fractional order parameter. The value α = 0.83 provides a fractional representation of temporal dependence in academic performance. The theoretical interpretation is based on the possibility that previous learning experiences retain a gradually declining influence on subsequent performance. The Caputo formulation represents this dependence through its memory kernel, which assigns different weights to previous states. The estimated α therefore describes the degree of fractional temporal dependence in the empirical model. It should not be interpreted as direct evidence that human learning universally follows a power law, nor as a direct psychological measure of memory.

The empirical results also highlight the importance of Educational support and learning behavior variables in improving student achievement. Family educational support and weekly study time show positive effects on academic performance within the fractional framework, indicating that sustained engagement in learning activities contributes to ongoing academic improvement. These findings agree with the extensive literature emphasizing the importance of parental involvement in educational success. Meta-analytic evidence consistently demonstrates that parental engagement is positively associated with students’ academic achievement, particularly when parents maintain high educational expectations and actively support learning at home.

Attendance behavior also emerges as an important determinant of academic performance. The negative association between student absences and final academic achievement suggests that regular participation in classroom activities remains essential for effective learning. This finding is consistent with previous research demonstrating that sustained class attendance contributes positively to academic success because students benefit from continuous exposure to instruction, classroom interaction, and collaborative learning opportunities.

Previous academic performance remains the strongest predictor of final achievement in both the benchmark and fractional models. The significant effects of first- and second-period grades indicate that learning outcomes accumulate progressively throughout the academic year. Within the proposed fractional framework, this persistence is interpreted as evidence of educational memory, whereby earlier academic achievements continue to influence subsequent learning. Rather than treating previous grades merely as control variables, the fractional specification recognizes them as indicators of accumulated educational capital that shapes future performance.

The robustness analysis further strengthens these conclusions by demonstrating that the estimated relationships remain stable across alternative fractional-order specifications. The consistency of coefficient signs, parameter magnitudes, and model performance measures indicates that the principal findings are not sensitive to moderate changes in the assumed degree of educational memory. Such stability increases confidence in the reliability of the proposed framework and suggests that the observed relationships reflect persistent characteristics of the educational process rather than model-specific artifacts.

From a practical perspective, the findings suggest that educational policies should emphasize sustained interventions rather than short-term corrective measures. School study support, structured learning assistance, regular attendance monitoring, and family engagement are unlikely to produce immediate improvements when implemented in isolation. Instead, their effectiveness appears to increase through continuous application over successive learning periods. This interpretation aligns with contemporary educational psychology, which emphasizes long-term student engagement, cumulative learning experiences, and supportive educational environments as key determinants of academic success.

An important methodological limitation concerns the dataset’s temporal structure. Only three academic assessments, G1, G2, and G3, are available within one academic year. These assessments provide an ordered sequence of academic performance, but they do not provide a sufficiently dense longitudinal trajectory to reliably identify a general long-memory process. In the present analysis, G1 and G2 represent the available academic history in the fractional component leading to G3. Nevertheless, the fractional order parameter is estimated from only two academic transitions and therefore requires cautious interpretation. The estimated value of 0.83 should not be generalized as definitive evidence of long-term educational memory. Future research should use datasets with substantially more repeated assessments for each student across multiple semesters or academic years. Such data would permit more reliable estimation and validation of fractional temporal dependence. The formal test against α = 1 indicates that the estimated fractional order differs significantly from the conventional first-order specification. Nevertheless, this statistical difference should be interpreted within the limitations of the three assessment periods and should not be treated as definitive evidence of a general long memory process.

The sensitivity analysis also assesses the potential overlap between previous academic performance and the fractional memory representation. When G1 and G2 are removed as conventional regression predictors, model performance decreases substantially. RMSE increases from 0.87 to 1.21, MAE increases from 0.66 to 0.91, and adjusted R^2^ decreases from 0.886 to 0.781. At the same time, the estimated fractional order remains within the admissible interval and decreases from 0.83 to 0.79. These results indicate that previous academic performance contains substantial information about G3. However, because G1 and G2 are also required to construct the discrete fractional memory term, the exclusion analysis does not separate fractional memory from prior academic performance. The results therefore provide evidence of sensitivity to the direct inclusion of previous grades, but they should not be interpreted as definitive evidence that the fractional memory effect is independent of prior academic achievement. Because the fractional memory term is constructed from the three available academic assessments, complete exclusion of G1 and G2 from the empirical fractional specification is not possible without changing the definition of the fractional model. Consequently, the present sensitivity analysis evaluates the effect of excluding previous grades as conventional predictors, while their information remains embedded in the discrete fractional memory term.

The analysis does not include several potentially relevant demographic and family structure variables, such as age, sex, family size, and parental status, because these variables were not included in the final model specification. The reported RMSE, MAE, adjusted R^2^, AIC, and BIC are based on the same observations used for model estimation. Therefore, they describe in-sample model fit and relative model adequacy rather than out-of-sample predictive performance. No training-testing split, cross-validation, bootstrap validation, or external validation is performed. Future studies should evaluate the framework using independent test samples or repeated longitudinal datasets. Inference for the nonlinear fractional order parameter is based on the local asymptotic covariance approximation. Bootstrap or profile likelihood procedures may provide additional robustness for inference with larger longitudinal datasets and should be considered in future studies. The present analysis does not include formal residual diagnostics, heteroscedasticity tests, influence diagnostics, or calibration analysis. In addition, the final grade is bounded on the observed 0–20 scale, while several explanatory variables are ordinal. The linear specification therefore represents a practical approximation to the observed grade scale rather than a fully specified bounded outcome model. Future analyses with larger, more detailed longitudinal datasets should examine these issues. The available data also do not permit direct empirical identification of the underlying functional form of memory decay. The fractional kernel is therefore treated as a theoretically motivated model specification rather than as evidence that academic learning necessarily follows a universal power law.

Additional limitations concern model diagnostics and functional form. The present analysis does not include formal residual, heteroscedasticity, influence, or calibration diagnostics. Multicollinearity is assessed through pairwise correlations rather than VIF or condition indices. The strong correlation between G1 and G2 therefore requires cautious interpretation of their estimated coefficients. Future studies should incorporate more extensive diagnostic procedures and alternative specifications for bounded academic outcomes.

## 5 Conclusion

This study proposed a fractional dynamic framework to examine the evolution of student academic performance in relation to educational support and learning behavior variables, using micro-level education data from 649 Portuguese secondary school students. Unlike conventional educational performance models, the proposed approach incorporated educational memory through a fractional-order formulation, recognizing that academic achievement is influenced by current Educational support and learning behavior variables as well as accumulated prior learning experiences. The analytical framework integrated conceptual modeling, fractional dynamics, empirical estimation, and robustness assessment to provide a comprehensive evaluation of student learning outcomes.

The empirical findings demonstrated that the proposed fractional dynamic model outperformed the benchmark educational performance model across multiple evaluation criteria. The benchmark model explained 85.2% of the variation in student academic performance, whereas the fractional specification increased the adjusted coefficient of determination from 0.849 to 0.886 and reduced the in-sample RMSE and MAE. The root mean square error decreased from 1.04 to 0.87, and the mean absolute error declined from 0.81 to 0.66. Similarly, the Akaike Information Criterion and Bayesian Information Criterion decreased from 2150.0 and 2213.0 to 2018.7 and 2088.4, respectively. These improvements indicate that incorporating educational memory provides a more accurate representation of the learning process than the conventional integer-order specification.

The estimated fractional order parameter of 0.83 indicates temporal dependence within the three academic assessment periods represented in the dataset. Given the limited number of observations, this result should be interpreted as evidence of temporal dependence within the observed assessment structure rather than as definitive evidence of a general long memory process. This finding suggests that learning outcomes develop progressively through the accumulation of previous educational experiences rather than through isolated instructional activities. Among the educational Explanatory variables, family educational support, weekly study time, school study support, and additional paid academic assistance were positively associated with academic achievement, whereas student absences and previous academic failures were negatively associated. Previous academic performance remained the strongest determinant of final achievement, thereby reinforcing the importance of cumulative learning in educational development. The robustness analysis further confirmed that these relationships remained stable across alternative fractional-order specifications, indicating that the empirical findings were not sensitive to moderate changes in the degree of educational memory.

The study contributes to the educational psychology literature by introducing fractional dynamic modeling as an alternative framework for analyzing student learning processes. The proposed approach extends existing educational performance models by explicitly incorporating persistence and memory dependence into the estimation process. From a practical perspective, the findings suggest that educational policies should prioritize continuous rather than short-term interventions. Sustained family engagement, regular attendance monitoring, structured academic support, and consistent study habits appear to produce greater improvements in student achievement when their cumulative effects are considered over successive learning periods. Consequently, educational institutions should adopt long-term intervention strategies that reinforce learning throughout the academic year instead of relying primarily on corrective measures implemented after academic difficulties have already emerged.

Despite these contributions, several limitations should be acknowledged. The empirical analysis was conducted using observations from Portuguese secondary schools, which may limit the generalizability of the findings to different educational systems and cultural contexts. In addition, the representation of educational memory was based on the sequential academic assessments available within the dataset rather than long-term longitudinal observations. Future research should therefore validate the proposed framework using panel datasets that follow students across multiple academic years and investigate alternative fractional operators and numerical solution techniques. The incorporation of additional psychological constructs, including academic motivation, self-efficacy, resilience, and student engagement, may also provide further insight into the mechanisms through which educational memory influences academic performance. Such extensions would strengthen the applicability of fractional dynamic modeling and contribute to the development of more effective evidence-based Educational support and learning behavior variables.

## Data Availability

The original contributions presented in the study are included in the article/supplementary material, further inquiries can be directed to the corresponding author.
